# Retraction: Neuroprotective effects of ginsenoside-Rg1 against depression-like behaviors via suppressing glial activation, synaptic deficits and neuronal apoptosis in rats

**DOI:** 10.3389/fimmu.2024.1437785

**Published:** 2024-06-03

**Authors:** Frontiers Editorial Office

Please, find the retraction statement below:

Cuiqin Fan, Qiqi Song, Peng Wang, Ye Li, Mu Yang, Shu Yan Yu (2018). Neuroprotective effects of ginsenoside-Rg1 against depression-like behaviors via suppressing glial activation, synaptic deficits and neuronal apoptosis in rats. Frontiers in Immunology 9:2889. doi: 10.3389/fimmu.2018.02889.

“Following publication, concerns were raised regarding the integrity of the images in the article. The authors failed to provide a satisfactory explanation during the internal investigation conducted by the Research Integrity Department, which was in accordance with Frontiers’ policies. Given these concerns Frontiers no longer have confidence in the findings presented in the article.

This retraction was approved by the Chief Executive Editor of Frontiers. The authors received a communication regarding the retraction”.

